# The genome of *Anoplarchus purpurescens* (Stichaeidae) reflects its carnivorous diet

**DOI:** 10.1007/s00438-023-02067-5

**Published:** 2023-09-10

**Authors:** Ninh Le, Joseph Heras, Michelle J. Herrera, Donovan P. German, Lisa T. Crummett

**Affiliations:** 1 Life Sciences Concentration, Soka University of America, Aliso Viejo, CA 92656 USA; 2 Department of Biology, California State University, San Bernardino, CA 92407 USA; 3 Department of Ecology and Evolutionary Biology, University of California, Irvine, CA 92697 USA; 4 Department of Epigenetics and Molecular Carcinogenesis, The University of Texas M.D. Anderson Cancer Center, Houston, TX 77030 USA

**Keywords:** Genomics, Gene copy number, Nutrition, Digestive enzyme, Long read, Short read, Feeding ecology

## Abstract

Digestion is driven by digestive enzymes and digestive enzyme gene copy number can provide insights on the genomic underpinnings of dietary specialization. The “Adaptive Modulation Hypothesis” (AMH) proposes that digestive enzyme activity, which increases with increased gene copy number, should correlate with substrate quantity in the diet. To test the AMH and reveal some of the genetics of herbivory vs carnivory, we sequenced, assembled, and annotated the genome of *Anoplarchus purpurescens*, a carnivorous prickleback fish in the family Stichaeidae, and compared the gene copy number for key digestive enzymes to that of *Cebidichthys violaceus*, a herbivorous fish from the same family. A highly contiguous genome assembly of high quality (N50 = 10.6 Mb) was produced for *A. purpurescens*, using combined long-read and short-read technology, with an estimated 33,842 protein-coding genes. The digestive enzymes that we examined include pancreatic α-amylase, carboxyl ester lipase, alanyl aminopeptidase, trypsin, and chymotrypsin. *Anoplarchus purpurescens* had fewer copies of pancreatic α-amylase (carbohydrate digestion) than *C. violaceus* (1 vs. 3 copies). Moreover, *A. purpurescens* had one fewer copy of carboxyl ester lipase (plant lipid digestion) than *C. violaceus* (4 vs. 5). We observed an expansion in copy number for several protein digestion genes in *A. purpurescens* compared to *C. violaceus*, including trypsin (5 vs. 3) and total aminopeptidases (6 vs. 5). Collectively, these genomic differences coincide with measured digestive enzyme activities (phenotypes) in the two species and they support the AMH. Moreover, this genomic resource is now available to better understand fish biology and dietary specialization.

**Supplementary Information:**

The online version contains supplementary material available at 10.1007/s00438-023-02067-5.

## Introduction

Comparing the genomes of closely related animals that have evolved different specializations offers opportunities to understand how differences in their physiology are attributed to differences on the genomic level. Whether it is epistatic interactions of suites of genes affecting phenotypes (e.g., Chen et al. 2022), changes in gene copy number impacting gene expression, which in turn affects enzyme activity levels (Axelsson et al. 2013; Heras et al. 2020; Perry et al. 2007), or molecular convergence of genes underlying unique phenotypic traits (Protas et al. 2005), genomic evidence of adaptation to environmental variables abound in the comparative genomics literature (Fan et al. 2020; Lamichhaney et al. 2015; Taylor et al. 2021; Yuan et al. 2018). As the nutrient supply organ that interfaces directly with items consumed from the environment, the digestive tract has become an attractive system in which to examine how animals can specialize on specific resources (Brun et al. 2020; Karasov and Douglas 2013). But how does dietary specialization impact an animal’s genome? With whole genome sequencing becoming more affordable and genomic analyses becoming more accessible, we are now better equipped to explore genomic adaptations to dietary specializations such as carnivory or herbivory (e.g., Axelsson et al. 2013; Heras et al. 2020; Wang et al. 2015).

Depending on the approach, one can start at the genomic level, find areas of a genome that may be under selection, and then scale up to discern what traits are impacted by the molecular changes identified (Axelsson et al. 2013; Yuan et al. 2018). Conversely, one can take a well-known system with well-studied physiological and biochemical processes and dig down to the genetic level to identify the underpinnings of specific phenotypes. This latter approach was done successfully in a recent examination of the herbivorous fish, *Cebidichthys violaceus* (Heras et al. 2020). Years of ecological, physiological, and biochemical data (Fris and Horn 1993; Gawlicka and Horn 2006; German et al. 2016; German et al. 2004; German et al. 2015; Horn et al. 1986; Kim et al. 2014) were used to make a priori predictions about how this herbivore is able to thrive on an algal diet. A detailed genomic analysis of *C. violaceus* largely confirmed these predictions and provided the genetic underpinnings of known herbivorous phenotypes (Heras et al. 2020). For instance, elevated amylolytic and lipolytic activities in the guts of the fish were attributed to increased gene copy number coding for those proteins.

In the context of digestive specialization, it is important to note that digestion is a chemical process, and the agents of that process are digestive enzymes. Thus, digestive enzymes play a crucial role in digestion and have been studied extensively in the context of diet (e.g., Brun et al. 2020; Vonk and Western 1984; Karasov and Douglas 2013; German et al. 2015, 2016; Schondube et al. 2001; Skea et al. 2005, 2007). The Adaptive Modulation Hypothesis (AMH) posits that digestive enzyme activity should correlate with substrate quantity in an animal's diet because protein synthesis would be wasted on enzymes targeting less abundant substrates (Karasov 1992; Karasov and Martínez del Rio 2007). To target abundant substrates, digestive enzyme activity can increase with increased expression level of the enzyme genes (Choi and Yamazaki 1994; Cockell et al. 1989; Gawlicka and Horn 2006; German et al. 2016; Howard et al. 1989; Ma et al. 2004; Wiebe et al. 2007), increased gene copy number of the enzyme genes, which increases expression (Axelsson et al. 2013; German et al. 2016; Gout et al. 2010; Qian and Zhang 2014; Springer et al. 2010), or expression of additional enzyme isoforms or gene variants (German et al. 2016). For instance, humans from agrarian backgrounds and domestic dogs (which have been consuming grains for nearly as long as humans) have expanded gene copy number of amylase genes, which correlates with increased amylase expression and higher enzymatic activity against starch (Axelsson et al. 2013; Perry et al. 2007). Similarly, in an examination of amylase genes and enzyme activity in stichaeid fishes, the herbivorous *C. violaceus* was found to have an extra copy of the amylase gene, elevated expression of amylase genes, and elevated amylase activity in comparison to carnivorous stichaeids (German et al. 2016).

**Fig. 1:**
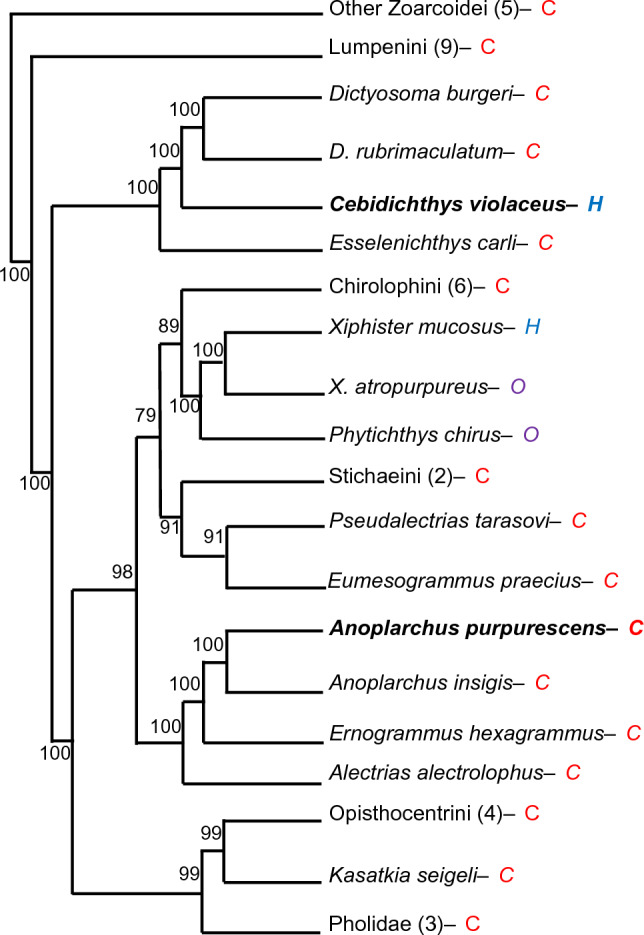
Summarized phylogenetic relationships of the family Stichaeidae based on 2100 bp of *cytb*, *16 s*, and *tomo4c4* genes (Kim et al. 2014). Bayesian posterior probabilities indicated on nodes are from Kim et al. (2014). Species used in this study bolded. *H* = herbivory, *O* = omnivory, *C* = carnivory. Numbers in parentheses show number of taxa evaluated at that branch

In this study, we set out to test predictions anchored in the AMH for how gene copy numbers for specific digestive enzymes will differ between a carnivorous fish, *Anoplarchus purpurescens*, and a sympatric herbivorous fish, *C. violaceus*, both in the family Stichaeidae (German and Horn 2006; Kim et al. 2014; Fig. 1). *Cebidichthys violaceus* and *A*. *purpurescens* represent separate intertidal invasions within the family Stichaeidae, where herbivory evolved in the former, but not the latter (Heras et al. 2020; Herrera et al. 2022; Fig. 1). The geographic range of *A. purpurescens* overlaps significantly with that of *C. violaceus*, with *A. purpurescens* extending from southern California to the Aleutian Islands to the north (Stoddard 1985). Based on the AMH, we predicted that, in comparison with the herbivorous *C. violaceus*, the carnivorous *A. purpurescens* will exhibit a reduction in gene copy number for enzymes that digest carbohydrates (carbohydrases), an expansion in gene copy number for enzymes associated with animal lipid digestion (triacylglycerol lipase) and/or a reduction in gene copy number for enzymes associated with plant lipid digestion (carboxyl ester lipase). We also predicted an expansion of gene copy number in *A. purpurescens* for one or more enzymes associated with protein digestion (proteases) that would correlate with increased dietary protein concentration and concomitant proteolytic activity in *A. purpurescens* relative to *C. violaceus* (German et al. 2004, 2015). This comparison will help uncover genomic differences associated with herbivory and carnivory in the family Stichaeidae, and vertebrates more broadly, and may have broad implications for aquaculture and fisheries management. We used long- and short-read technology to sequence, assemble, and annotate the genome of *A. purpurescens*, yielding even better genome assembly metrics (e.g., N50 of 10.6 Mb) than the *C. violaceus* genome (N50 of 6.7 Mb; Heras et al. 2020). Currently, there is little known about genome size or chromosome number for *A. purpurescens*, which we assumed would be similar to *C. violaceus*.

## Materials and methods

### Collection and preparation

One individual of *A. purpurescens* (85 mm standard length) was collected in January 2021 from San Simeon, California (35.6525°N, 121.2417°W). The individual was euthanized in tricaine methanesulfonate (Syndel, Ferndale, Washington, USA; 1 g l^−1^), dissected for internal organ removal, decapitated and preserved in liquid nitrogen. Fish handling from capture to euthanization was conducted under approved protocol 2021–012 of the Institutional Animal Care and Use Committee (IACUC) at the University of California, Irvine. Genomic DNA was extracted from 250 mg of skin and muscle tissue using a Qiagen Blood & Cell Culture DNA Midi Kit (Qiagen, Redwood City, California, USA), following the manufacturer’s instructions. Following extraction, the DNA samples were sheared and separated into high molecular weight DNA fragments by a pulse field electrophoresis. We used Pacific Biosciences (PacBio) and Illumina platforms for sequencing. For PacBio sequencing, genomic DNA was size-selected, with a 15 kb size cut-off, using a BluePippin Size Selection System. A PacBio Sequel II was used to sequence one Single Molecule Real Time (SMRT) cell, which can produce up to 100 Gb of sequence data, and in our case, 74 Gb. Additionally, from the same gDNA extraction, a multiplex gDNA-Seq Illumina sequencing library was prepared from size-selected fragments ranging from 500 to 700 bp, and sequenced on two lanes on an Illumina HiSeq 4000, which resulted in short reads (100 bp paired-end). All genomic sequencing was completed at the University of California, Irvine (UCI) Genomics Research and Technology Hub (GRTH) following Heras et al. (2020).

### Assembly of Illumina short reads and PacBio long reads

Computational and bioinformatics analyses were all conducted on the High-Performance Computing (HPC) Cluster at UCI. Paired-end Illumina sequence data was examined for quality control using FastQC version 0.11.9 (Andrews 2010). Trimming and adapter removal was performed by BBDuk version 38.87 with the following parameters: ref = adapters, phix ktrim = r mink = 11 hdist = 1 qtrim = rl trimq = 10 (Bushnell 2014; Supplemental Fig. S1). Genome size and heterozygosity percentage were estimated from unassembled Illumina reads with GenomeScope1.0 (Marçais and Kingsford 2011; Vurture et al. 2017) and ploidy was confirmed with Smudgeplot version 0.1.3 (Ranallo-Benavidez et al. 2020; Supplemental Fig. S1). The k-mer size was set to 21 for these programs. Illumina sequencing data were assembled with Platanus version 1.2.4 (Kajitani et al. 2014) with the following parameters: platanus assemble -t 24 -m 240 -k 17 -s 2; where initial k-mer size was set at 17 and step size was set to 2 (Supplemental Fig. S1, S2).

We conducted a PacBio SMRT Sequencing read-only assembly using Flye version 2.9-b1787 (Kolmogorov et al. 2019) with “scaffolding mode” on and “keep-haplotype” mode off (Supplemental Fig. S1).

### Combined hybrid assembly

To produce a hybrid assembly, we input the contigs generated by Platanus and the raw long-read sequencing data into DBG2OLC (Ye et al. 2016; Supplemental Fig. S1) with the following parameters: k 17 KmerCovTh 2 MinOverlap 150 AdaptiveTh 0.02 LD1 0 and RemoveChimera 1. To reduce the size of the data set, DBG2OLC “compressed” each long read into an ordered set of Platanus contigs that mapped to it (above certain thresholds set by AdaptiveTh and KmerCovTh). The cleaned compressed long reads were then used to construct a best overlap graph, from which backbone sequences were generated. Finally, a consensus module, Pbdagcon version 0.3 (Chin et al. 2013), was utilized with default parameters to align reads to each backbone to produce the polished final assembly (Supplemental Fig. S1).

We used the long-read assembly from Flye as the reference assembly and the hybrid assembly from DBG2OLC as the query assembly for Quickmerge v.1.0 (Chakraborty et al. 2016), which is both a meta-assembler and assembly gap filler program originally developed for long read assemblies (Supplemental Fig. S1). Quickmerge clusters contigs between the query and the reference assemblies based on their high confidence overlap (HCO), a metric score that quantifies how well a contig overlaps with another. Different parameters were tested until the most contiguous assembly was obtained with the following parameters: -hco 5, -c 1.5, -l 1,000,000, -ml 5000. We conducted a second round of Quickmerge using the output assembly from the first round of Quickmerge as the reference assembly, and the Flye assembly was used as the query assembly, with the following parameters: -hco 5, -c 1.5, -l 3,900,000, -ml 5000.

### Hybrid assembly polishing, purging, and repeat masking

We polished the *A. purpurescens* hybrid genome assembly (Supplemental Fig. S1) through two rounds of Arrow, executed through gcpp version 2.0.2 (https://github.com/PacificBiosciences/gcpp), where long reads were aligned to our quickmerge assembly by Pbmm2 version 1.4.0 (https://github.com/PacificBiosciences/pbmm2). We used purge_dups (Supplemental Fig. S1), which utilizes sequence similarity and read depth to resolve haplotigs and homologous chromosome overlaps (Guan et al. 2020). To mask repetitive elements, the final genome assembly was processed through Repeatmasker version 4.1.2 (Smit et al. 2015) with the parameters -e ncbi -pa 8 -species teleostei -s -xsmall, using the complete Dfam library (Supplemental Fig. S1).

### Hybrid assembly quality assessment

The genome size and the N50 value for the hybrid assemblies (Table 1) were computed using a Perl script (Bradnam et al. 2013), while the heterozygosity rates were estimated with a pipeline consisting of Burrows–Wheeler aligners version 0.7.8 (Li and Durbin 2009), SAMtools version 1.10 (Li et al. 2009), BCFtools version 1.14 (Li et al. 2009), and a Python script provided by Dr. John Bracht (Asalone et al. 2020; Supplemental Fig. S1). Heterozygosity estimates of assemblies were corroborated with analyses of spectra copy number plots generated by Merqury (Rhie et al. 2020) and Meryl version 1.3 (Walenz 2020), the latter providing 21-mer count histograms for the former (Supplemental Fig. S1, S2). The completeness of our *A. purpurescens* genome was evaluated using BUSCO (Benchmarking Universal Single Copy Orthologs) version 5.3.0, using the Vertebrata and Actinopterygii gene sets (Simão et al. 2015).

**Table 1:** Genome assembly programs, types of reads used, and statistics used to assemble the genome of *Anoplarchus purpurescens*

Assembly	Programs used	Reads used	Assembly size (bp)	Scaffold/Contig number	N50 value (bp)
1	Platanus	Illumina	667,076,046	2,599,093	1,207
2	DBG2OLC, Blasr, pbdagcon	Illumina PacBio	598,594,787	976	1,980,038
3	Flye	PacBio	582,412,549	1,331	3,956,814
4	Quickmerge (round 1)	Illumina PacBio	593,045,980	732	6,841,580
5	Quickmerge (round 2)	Illumina PacBio	585,237,394	1,210	10,452,245
6	Pbmm2, Arrow	Illumina PacBio	586,262,929	1,210	10,464,639
7	Purge_dup	Illumina PacBio	567,389,083	489	10,617,371

### Genome synteny analyses

After concatenating contigs less than 1 MB into one contig per genome, we ordered the genome of *A. purpurescens* against that of *C. violaceus* using SyMAP v5.2.0 (Synteny Mapping and Analysis Program; Soderlund et al. 2011). CIRCOS and synteny blocks between the two species were computed with SyMAP v5.2.0 at default parameters.

### Structural gene annotation

We used the BRAKER2 pipeline (Altschul et al. 1990; Brůna et al. 2021; Camacho et al. 2009; Hoff et al. 2016, 2019; Stanke et al. 2006, 2008) to perform structural gene annotations on the genomes of both *A. purpurescens* and *C. violaceus*, the latter sequenced and assembled by Heras et al. (2020). The Vertebrata section of the OrthoDB database (Kriventseva et al. 2019) was used to generate protein hints by BRAKER2, and was processed by the ProHint pipeline (Brůna et al. 2020; Buchfink et al. 2015; Gotoh et al. 2014; Iwata and Gotoh 2012; Lomsadze et al. 2005). RNA sequence data for *A. purpurescen*s and *C. violaceus* were taken from Herrera et al. (2022) and Heras et al. (2020) respectively. The RNA sequence data was trimmed by TrimGalore version 0.6.6 (https://github.com/FelixKrueger/TrimGalore). We aligned the RNA-seq data against their respective genomes using HISAT2 version 2.1.0 (Kim et al. 2019), which provided spliced alignments for BRAKER (Barnett et al. 2011; Li et al. 2009). The alignments were utilized by GeneMark-ET to generate a training gene set for AUGUSTUS (version 3.5; Lomsadze et al. 2014). We used the RNA-seq data of the liver, mid-intestine and pyloric caeca tissues of wild-type *A. purpurescens* (Herrera et al. 2022), and that of the spleen, mid-intestine, gonads, pyloric caeca, heart, brain, liver, proximal intestine, and gill tissues of *C. violaceus* (Heras et al. 2020). The unassembled RNA-seq data of both species were included in all replicates, if replicates were available, to retain and increase coverage information of each splice site, optimizing GeneMark-ET performance (Hoff et al. 2019). The final genome annotation was uploaded as project PRJNA950117 at NCBI (https://www.ncbi.nlm.nih.gov/bioproject/).

### Functional gene annotation

The transcripts predicted by AUGUSTUS, from the BRAKER2 pipeline, were functionally annotated by BLAST2GO from OmicsBox version 2.2.4 (Götz et al. 2008). Within BLAST2GO, we used blastx-fast, with default parameters, to search the Vertebrata subset of the non-redundant protein sequence database, version 5 (Götz et al. 2008). Gene identities were derived from BLAST2GO consensus descriptions.

### Creation of gene synteny maps among fish species

We compared the genomic regions surrounding specific digestive enzymes, among *A. purpurescens*, *C. violaceus*, and several non-stichaeid fish, including *Danio rerio*, *Oryzias latipes, Gasterosteus aculeatus*, and *Oreochromis niloticus*. Multiple sequence alignments of specific genomic regions, among six fish species, were performed with MUSCLE version 3.8.425 (Madeira et al. 2022), and phylogenetic trees were made with 1,000 bootstrap replicates using PhyML 3.0, where AIC helped to determine the best model (Guindon et al. 2010; Lefort et al. 2017).

## Results

### Quality and coverage of sequence data from Illumina and PacBio platforms

From one PacBio SMRT cell sequencing, we generated 74 Gb long reads, with approximately 70X coverage. Illumina generated 36 Gb of 100 bp paired-end reads, approximately 24X coverage. The quality of Illumina reads was excellent, with reads having an average QC score of 34–36 in all positions, which translates to a base call accuracy of 99.97%.

### Estimated characteristics of the *A. purpurescens* genome

At a k-mer size of 21, GenomeScope estimated, with high confidence, the haploid genome size of *A. purpurescens* to be 538,951,370 bp, which is similar to other fish genomes (Heras et al. 2020). There were an estimated 33,842 protein-coding genes in the genome. GenomeScope estimated the heterozygosity percentage of the *A. purpurescens* genome to be 0.879% (Supplemental Fig. S2). The diploid nature of the *A. purpurescens* genome was confirmed by Smudgeplot (Ranallo-Benavidez et al. 2020; Supplemental Fig. S3). RepeatMasker identified 32.02% of the genome assembly as repetitive sequences, including 4.48% as retroelements, and 8.75% as DNA transposons (Supplemental Table S1).

### Quality of the final assembly

Utilizing both the Illumina contigs and long reads from PacBio SMRT sequencing, the hybrid assembly produced a more contiguous assembly than using the short reads, alone (N50 = 1.99 Mb vs. N50 = 1,207 bp; Table 1). The PacBio long-read assembly, using Flye, yielded an N50 value that was more than double the N50 value from the original hybrid assembly (N50 = 3.96 Mb vs. 1.99 Mb; Table 1). Two rounds of merging the hybrid assembly with the long-read assembly produced a highly contiguous assembly with the highest N50 value (10.46 Mb) and 1210 scaffolds (Table 1). Reducing the number of scaffolds and contigs, via purging, increased the N50 value further to 10.62 Mb and decreased the scaffold count to 489 (Table 1). Purging also brought the hybrid genome assembly size (567 Mb) closer to the haploid genome size estimated by GenomeScope, 538 Mb (Table 1). BUSCO showed that the final hybrid genome assembly is 97% complete with 33,862 protein-coding genes.

### Digestive enzyme gene copy number in *A. purpurescens* vs. *C. violaceus*

**Fig. 2:**
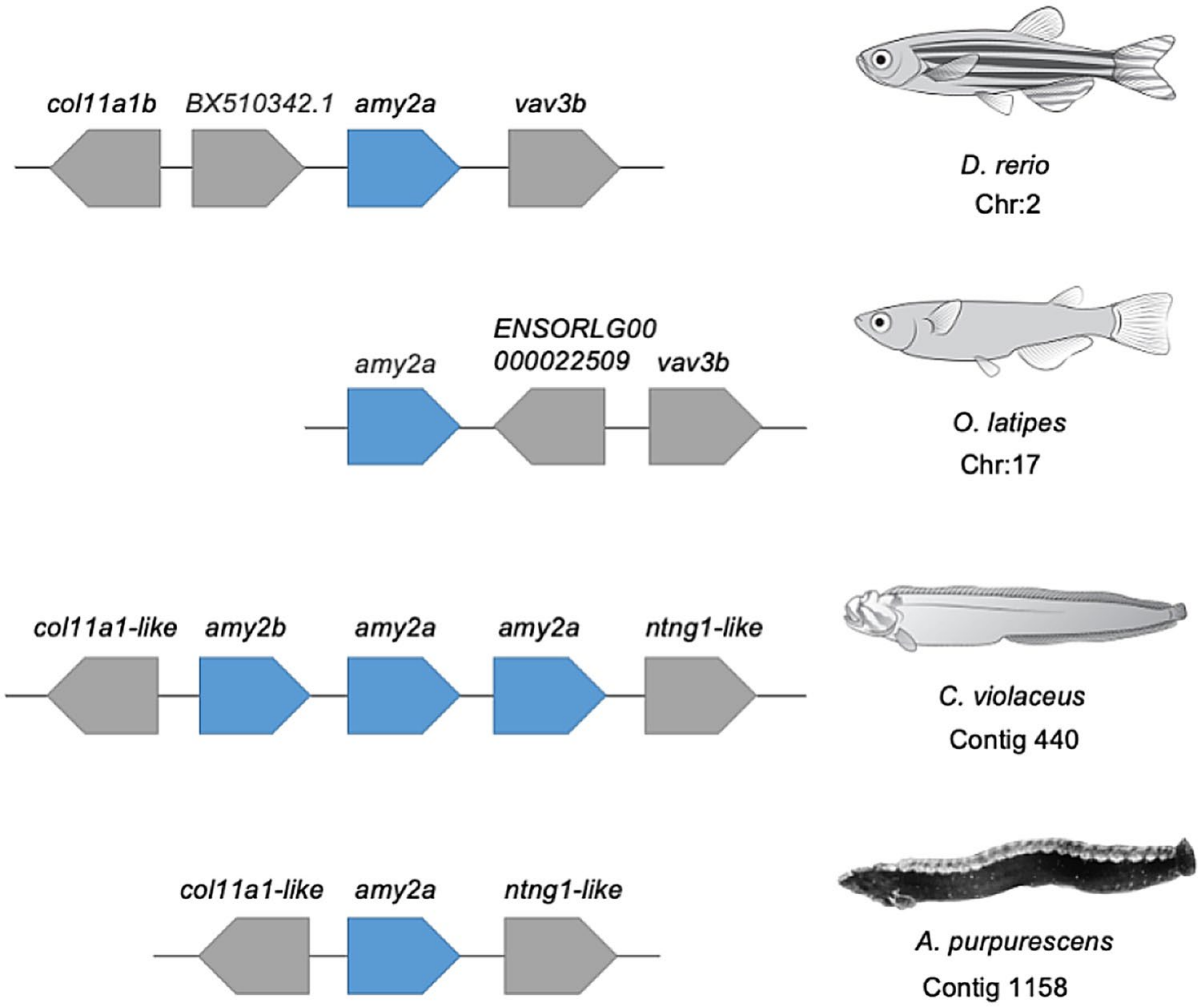
Synteny map for pancreatic α-amylase genes (*amy2*) from *D. rerio, O. latipes*, *C. violaceus*, and *A. purpurescens.* See Supplemental Table S2 for information on genetic resources for each species

**Fig. 3:**
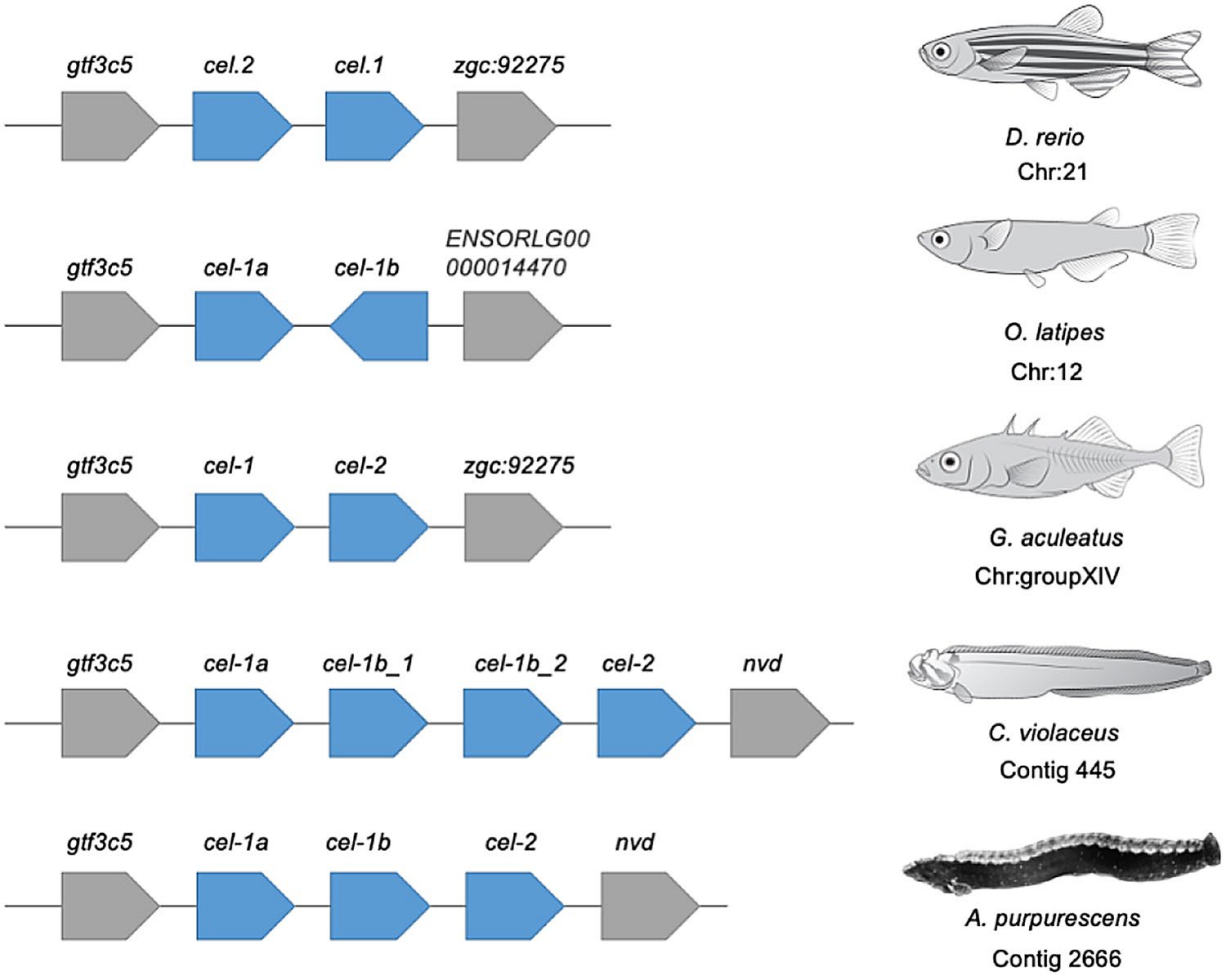
Synteny map for carboxyl ester lipase genes (*cel*) from *D. rerio, O. latipes*, *G. aculeatus*, *C. violaceus*, and *A. purpurescens.* See Supplemental Table S2 for information on genetic resources for each species

**Fig. 4:**
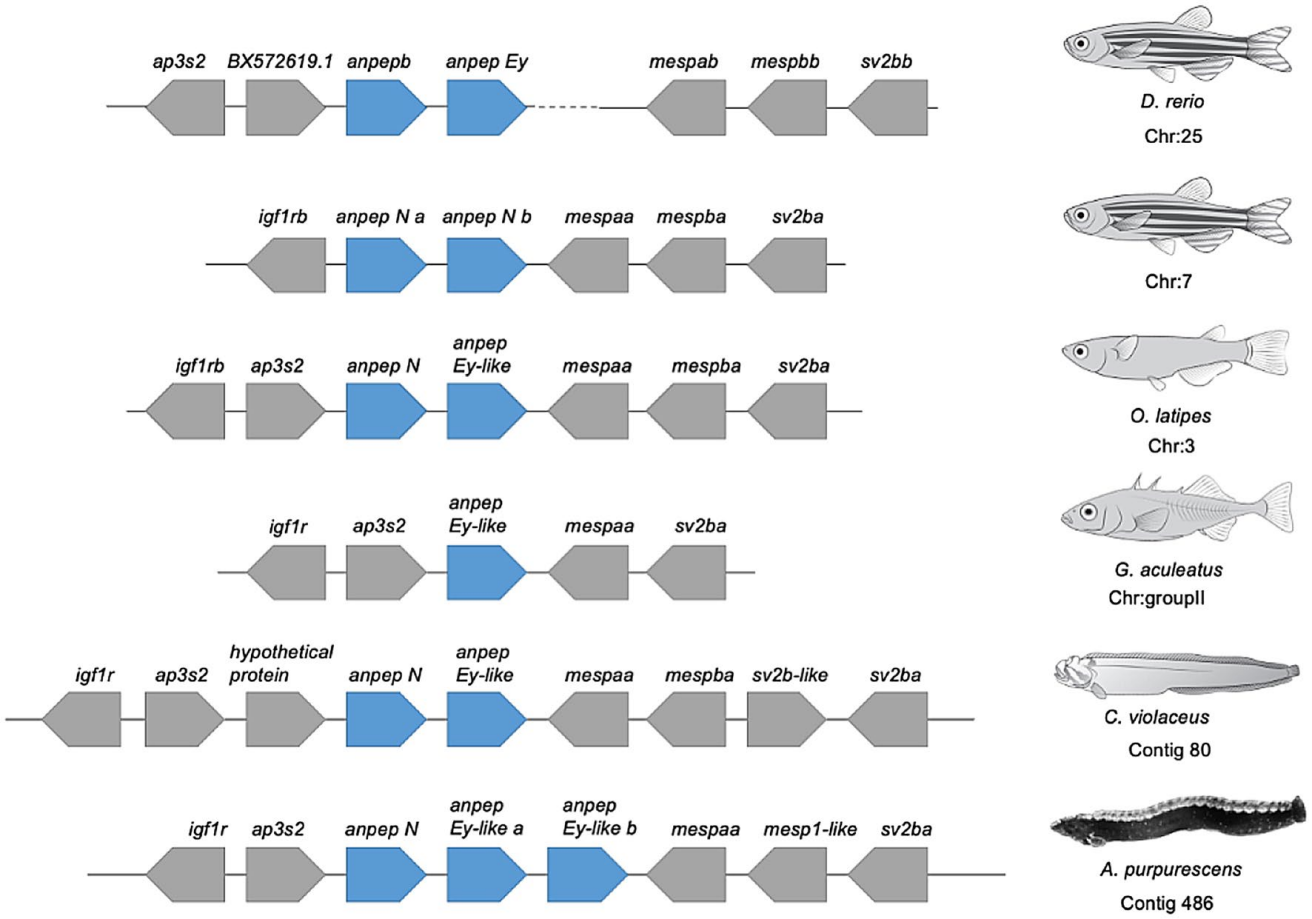
Synteny map for aminopeptidase N and Ey-like genes (*anpep N* and *anpep Ey-like*) from *D. rerio, O. latipes*, *G. aculeatus*, *C. violaceus*, and *A. purpurescens.* See Supplemental Table S2 for information on genetic resources for each species

**Fig. 5:**
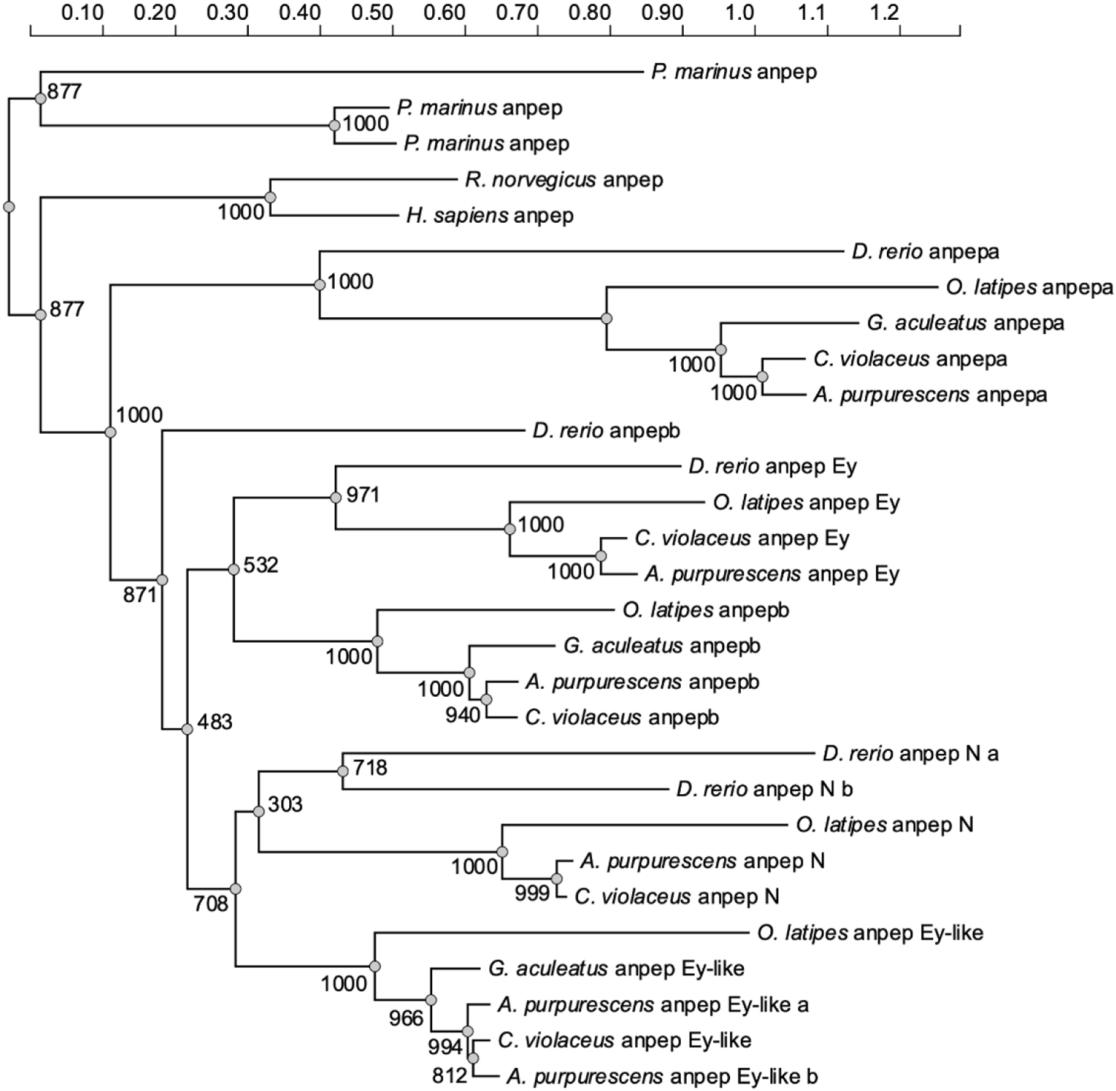
Phylogenetic relationship of alanyl aminopeptidase genes (*anpep*) in fishes (including *A. purpurescens*). A maximum likelihood (ML) tree was constructed with 1000 bootstrap replicates in PhyML v3.0 based on alanyl aminopeptidase sequences from *A. purpurescens*, *C. violaceus*, *G. aculeatus*, *O. latipes*, *D. rerio*, *Homo sapiens* and *Rattus norvegicus*. Alanyl aminopeptidase sequences from *Petromyzon marinus* were used as an outgroup. See Supplemental Table S2 for information on genetic resources for each species

**Fig. 6:**
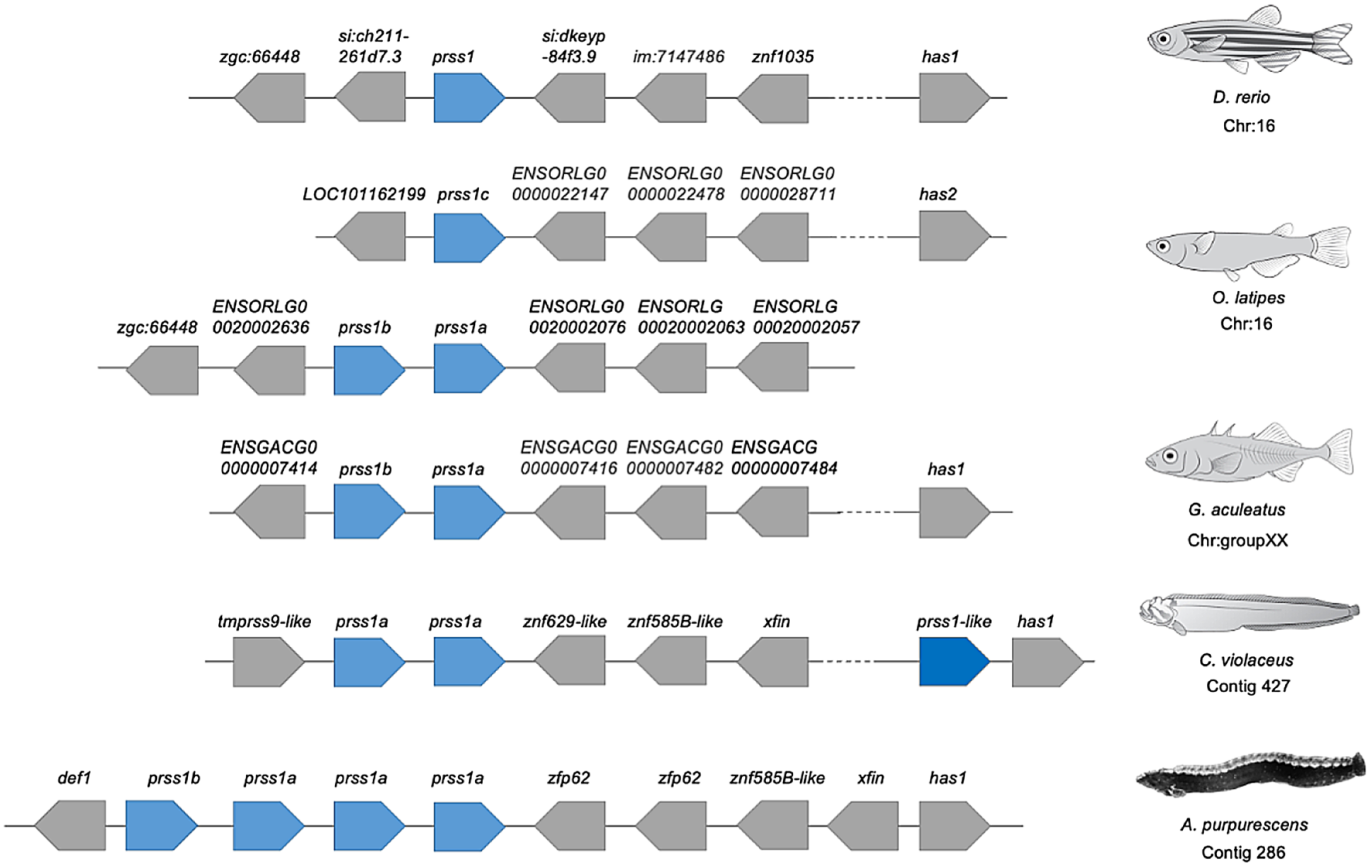
Synteny map for trypsin genes (*prss*) from *D. rerio, O. latipes*, *G. aculeatus*, *C. violaceus*, and *A. purpurescens*. See Supplemental Table S2 for information on genetic resources for each species

**Fig. 7:**
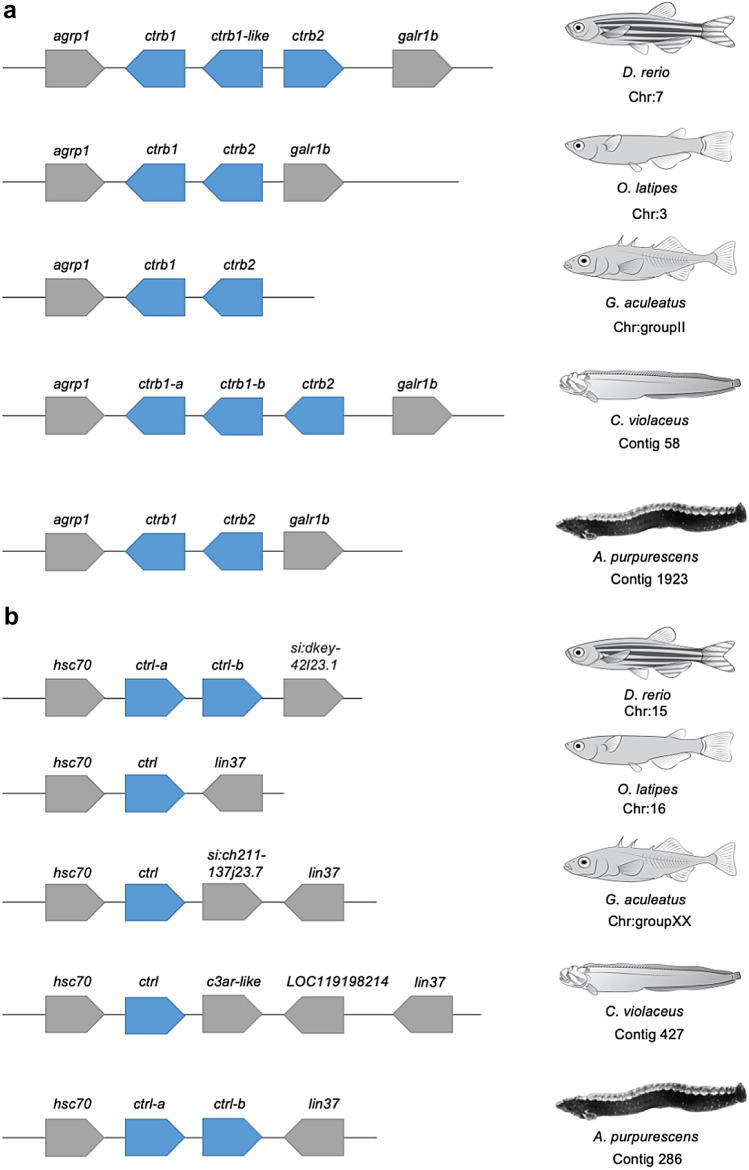
**a** Synteny map for chymotrypsin genes (*ctr*) from *D. rerio, O. latipes*, *G. aculeatus*, *C. violaceus*, and *A. purpurescens* **b** Synteny map for chymotrypsin-like genes (*ctrl*) from *D. rerio, O. latipes*, *G. aculeatus*, *C. violaceus*, and *A. purpurescens*. See Supplemental Table S2 for information on genetic resources for each species

The genomic comparisons of *A. purpurescens* and *C. violaceus* revealed highly syntenic genomes (Supplemental Fig. S4) with differences in gene copy number among key digestive enzyme genes. *A. purpurescens* has a reduced number of pancreatic α-amylase (*amy2*) genes compared to *C. violaceus* (1 vs. 3 copies; Table 2; Fig. 2); The AMY2 enzyme digests dietary starches (carbohydrates). *A. purpurescens* had a reduced number of carboxyl ester lipase (*cel*) genes compared to *C. violaceus* (4 vs. 5 copies; Table 2; Fig. 3 and Supplemental Fig. S5). The CEL enzyme efficiently digests plant lipids. Both species had one copy each of triacylglycerol lipase (*lipc*) and hepatic triacylglycerol lipase (*lipf*), which efficiently digests triacylglycerol, the main constituent of body fat in animals (Sahaka et al. 2020; Table 2). We observed an expansion in gene copy number for two proteolytic enzymes in *A. purpurescens* (Table 2). *A. purpurescens* had more copies of aminopeptidase Ey-like (2 vs. 1 copies; Table 2; Figs. 4 and 5) than *C. violaceus*, whereas *A. purpurescens* had more copies of trypsinogen (5 vs. 3 copies; Table 2; Fig. 6; Supplemental Fig. S8). While the total number of chymotrypsin genes is the same between *A. purpurescens* and *C. violaceus*, the species differ in the number of gene copies of chymotrypsin B1 (1 vs. 2 copies; Table 2; Fig. 7a) and chymotrypsin-like genes (2 vs. 1 copies; Table 2; Fig. 7b).

**Table 2:** Gene copy numbers of pancreatic α-amylase, carboxyl ester lipase, chymotrypsinogen, trypsinogen, alanyl aminopeptidases, gastric and hepatic triacylglycerol lipase in *Anoplarchus purpurescens* and *Cebidichthys violaceus*

Gene full name	Gene acronym	Gene copy number	
		*A. purpurescens*	*C. violaceus*
Pancreatic α-amylase	*amy*2	**1**	**3**
Carboxyl ester lipase (total)		**4**	**5**
Carboxyl ester lipase 1	*cel* 1	2	3
Carboxyl ester lipase 2	*cel* 2	1	1
Carboxyl ester lipase like	*cel-like*	1	1
Chymotrypsinogen (total)		**4**	**4**
Chymotrypsinogen B1	*ctr*b 1	1	2
Chymotrypsinogen B2	*ctr*b 2	1	1
Chymotrypsinogen-like	*ctrl*	2	1
Trypsinogen (total)		**5**	**3**
Trypsinogen 1	*prss 1*	4	2
Trypsinogen 2	*prss 2*	1	1
Aminopeptidase (total)		**6**	**5**
Aminopeptidase A	*anpep a*	1	1
Aminopeptidase B	*anpep b*	1	1
Aminopeptidase N	*anpep N*	1	1
Aminopeptidase Ey	*anpep Ey*	1	1
Aminopeptidase Ey-like	*anpep Ey-like*	2	1
Gastric triacylglycerol lipase	*lipc*	**1**	**1**
Hepatic triacylglycerol lipase	*lipf*	**1**	**1**

## Discussion

We assembled the genome for the carnivorous prickleback fish, *Anoplarchus purpurescens* with the intent of comparing it to that of the herbivorous *C. violaceus* so that we could observe potential dietary adaptations on the level of digestive enzyme genes. Matching with gene expression (Gawlicka and Horn 2006; German et al. 2016; Heras et al. 2020; Herrera et al. 2022; Kim et al. 2014) and digestive enzyme activity data (German et al. 2004, 2015) gathered in many previous studies of these fish species, we found support in the form of gene copy number differences in digestive enzyme genes that match with dietary-related phenotypes in these species. Hence, we provide clear support for the AMH (Karasov 1992; Karasov and Martinez del Rio 2007) on the genomic level to support previously measured phenotypes.

The N50 value of the *A. purpurescens* genome, at 10.6 Mb, makes it one of the most contiguous fish genomes in the literature (Lu and Luo 2020; Lu et al. 2020) (and see Supplemental Table S1 in Heras et al. 2020). The BUSCO score of 97% also illustrates a nearly complete assembly and annotation. The size of the *A. purpurescens* genome (539 Mb) shows that it is similar in size to other fish genomes spanning a deep diversity of fisheries and aquaculture species (Lu and Luo 2020), as well as those of *C. violaceus* and *Siniperca knerii* (Heras et al. 2020; Lu and Luo 2020; Lu et al. 2020). The success of this genome assembly further supports that merging multiple assemblies with Quickmerge (Chakraborty et al. 2016) can be done for species that are not model organisms (e.g., Baldwin-Brown et al. 2018; Heras et al. 2020). Moreover, the *A. purpurescens* genome is highly syntenic with that of *C. violaceus* (Supplemental Fig. S4), allowing us to make comparisons between these two closely related fish species with different diets (German et al. 2015; Herrera et al. 2022).

Based on the AMH (Karasov 1992; Karasov and Martínez del Rio 2007), we predicted that *A. purpurescens* would have fewer amylase (*amy2*) gene copies than *C. violaceus* as carnivorous fishes consume much less starch in their diet compared to herbivorous fishes and increased gene copy number often results in increased gene expression and increased protein activity (Axelsson et al. 2013; German et al. 2016; Gout et al. 2010; Heras et al. 2020; Perry et al. 2007; Qian and Zhang 2014). This prediction was supported (Fig. 2) and this finding is in line with the observation that *C. violaceus* has gut amylolytic activity that is more than an order of magnitude higher than that of *A. purpurescens*, reflecting the higher starch content in the diet of *C. violaceus* (German et al. 2004, 2015; Neighbors and Horn 1991). In general, carnivorous fishes have lower gut amylase activities than herbivorous or omnivorous fishes (Chakrabarti et al. 1995; Chan et al. 2004; Fernandez et al. 2001; Hidalgo et al. 1999; Sabapathy and Teo 1993). Moreover, the observation of elevated amylolytic activity in the guts of animals consuming more starch is well-known across nearly all animal clades (see German et al. 2016 for a detailed discussion).

Our prediction of fewer gene copies of carboxyl ester lipase (*cel*) in *A. purpurescens* compared to *C. violaceus* was supported (Fig. 3), whereas there was no difference in gene copy number for gastric triacylglycerol lipase and hepatic lipase between the two species (Table 2). Heras et al. (2020) confirmed that the *cel* genes are expressed in the gut and code for the carboxyl ester lipase (CEL) enzyme, which is the primary intestinal lipase in fishes and has broad specificity, especially toward algal and plant lipids (Li-Beisson et al. 2019; Murray et al. 2003; Olsen and Ringø 1997; Sæle et al. 2010). Although an animal-based diet contains more total lipids (Neighbors and Horn 1991), those lipids are more of the triacylglycerol variety (Sahaka et al. 2020). Plant material, although lower in lipid than animal tissue, is abundant in galacto- and betaine lipids (Kato et al. 1996; Li-Beisson et al. 2019; Sahaka et al. 2020), which CEL efficiently digests. Data for lipolytic activity against different lipid types is lacking for fishes, but herbivorous and omnivorous insects and mammals hydrolyze galactolipids at a higher rate than carnivores, whereas triacylglycerides are hydrolyzed at higher rates in carnivorous insects and mammals (Amara et al. 2010; Christeller et al. 2011). Animal material and plant material both contain phospholipids (German et al. 1996; Murray et al. 2003), and phospholipase gene copy number has not been found to vary among the fish species that have been examined thus far (Castro-Ruiz et al. 2021; Heras et al. 2020). While fishes may not possess the lipase diversity found in mammals (Murray et al. 2003; Olsen and Ringø 1997; Sæle et al. 2010; Tang et al. 2022), they do possess several *cel* genes that are worth investigating (Tang et al. 2022; Fig. 3, Supplemental Figs. S5 and S6). There are generally two *cel* loci in fishes: one that contains *cel*-1 and *cel*-2 (and their copies), and a different one for *cel*-like (Tang et al. 2022; Fig. 3, Supplemental Figs. S5 and S6). Each of these *cel* genes group separately in gene phylogenetic trees, showing that they have their own properties (Tang et al. 2022; Supplemental Fig. S6), although Tang et al. (2022) haphazardly named their *cel* genes as bile salt activated lipase (*bsal*) with random numbers, which we attempted to salvage based on the phylogenetic relationships of the genes themselves (Supplemental Fig. S6). Like *anpep*, the naming of *cel* genes in fishes deserves more attention. The extra copy of *cel* 1 in the *C. violaceus* genome could help explain the observation that total lipolytic activity (across the whole gut) in *C. violaceus* is more than twice that of *A. purpurescens* (German et al. 2004, 2015). However, more specific analyses are needed, including a pH stat method (Amara et al. 2010; Christeller et al. 2011; Sahaka et al. 2020), to differentiate between different types of lipase activities and to determine what dietary substrates the lipases in *C. violaceus* and *A. purpurescens* can hydrolyze.

Perhaps one of the most intriguing findings in this study is that *A. purpurescens* has only one additional copy of alanyl aminopeptidase (Figs. 4 and 5, Supplemental Fig. S6), compared to *C. violaceus.* This finding was surprising given that, on a per-gram tissue basis, the carnivorous *A. purpurescens* has approximately doubled the aminopeptidase activity in its gut compared to the herbivorous *C. violaceus* (German et al. 2004, 2015). The aminopeptidase enzyme hydrolyzes peptides prior to amino acid absorption in the intestine (Karasov and Douglas 2013). The additional copy of aminopeptidase in *A. purpurescens* is at the *anpep Ey-like* locus (Fig. 4), but there are five different copies of alanyl-aminopeptidase in each species (*anpep a*,* anpep b*,* anpep N*,* anpep Ey*,* anpep Ey-like*), that are also found in other fishes (Figs. 4 and 5, Supplemental Fig. S7). This motivated us to follow up on the phylogenetic analysis of *anpep* genes that Heras et al. (2020) first reported. The three ancestral vertebrate *anpep* genes in *Petromyzon marinus* are most similar to the *anpep N* gene in mammals, and in turn, these are sister to the fish aminopeptidases (with the limited number we analyzed; Fig. 5). The most ancestral teleost aminopeptidase is *anpep a*. In vertebrate evolution, there were two rounds of whole genome duplication (WGD), followed by a teleost-fish-specific WGD event (Christoffels et al. 2004; Glasauer and Neuhauss 2014; Kasahara 2007; Ohno 1970). Genes retained from WGDs are known as ohnologs (Ohno 1970). According to the website http://ohnologs.curie.fr, which predicts ohnologs, *anpep a* and *anpep b* are ohnologs from one of the vertebrate WGD events, whereas *anpep b* and *anpep N* are ohnologs from the teleost-fish-specific WGD (Heras et al. 2020; Kasahara 2007; Ohno 1970). There is similarity of the surrounding genes, in the respective loci, of these *anpep* genes (Heras et al. 2020). It appears that *anpep Ey* is a paralog of *anpep b*, whereas *anpep Ey-like* is a paralog of *anpep N* (Fig. 5, Supplemental Fig. S7). Each of these is found in other teleost fishes, with some variations (e.g., *G. aculeatus* apparently lacks *anpep N* and *D. rerio* lacks *anpep Ey-like*; Figs. 4 and 5, Supplemental Fig. S7; Heras et al. 2020). Each of these *anpep* genes shows strong gut expression in pricklebacks (Heras et al. 2020; Herrera et al. 2022). ANPEP enzyme activity in *A. purpurescens* should be explored in more detail to determine if the extra copy of *anpep Ey-like* is causing elevated enzymatic activity in this species, and/or some other *anpep* gene is being expressed at a higher level (e.g., Brun et al. 2021) and boosting ANPEP activity.

We have observed some inconsistencies with the number of, and naming of, vertebrate alanyl-aminopeptidases. What is known as *anpep N* in humans and other mammals is actually an ancestral vertebrate *anpep* that is more similar to those of *P. marinus* and *anpep a* in fishes. Therefore, what is termed “*anpep N”* in fishes is not an ortholog of *anpep N* in mammals. Moreover, *anpep Ey* and *anpep Ey-like* are not sister to one another. They are sister to *anpep b* and *anpep N*, respectively. What each of these ANPEP proteins does in the digestive process requires further investigation, and further, the naming of these genes and their resultant proteins needs to be given some attention. Dietary protein is an important nutrient for all animals (Brun et al. 2021; Horn 1989), and fishes have retained numerous *anpep* genes (and presumably enzymes) to ensure its digestion and absorption. A better understanding of aminopeptidase function, and perhaps distribution along the gut, could have implications for better aquaculture feed design (Tang et al. 2016). Moreover, aminopeptidases are also implicated in immune function of fishes and lepidopterans (Erşahin et al. 2005; Tang et al. 2016), and some of this *anpep* diversification could have function beyond digestion.

Further supporting our predictions, we found two additional copies of another protease, trypsinogen-1 (*prss 1*) in *A. purpurescens* compared to *C. violaceus* (Fig. 5, Supplemental Figs. S8 and S9). As with all proteolytic enzymes, trypsin (a pancreatic serine protease) is first synthesized as a zymogen that must be activated before it is able to perform its hydrolytic action on peptide bonds, and hence, the gene is for the zymogen, trypsinogen, instead of the active protease, trypsin (Voet and Voet 1995; Vonk and Western 1984). Most fishes appear to possess two copies of the *prss 1* gene, and *C. violaceus* is no different. However, *A. purpurescen*s has four copies of *prss 1* in tandem. *C. violaceus* does have a trypsin-like gene further down on the same contig, and this appears to be sister to all of the *prss 1* genes we examined (Supplemental Figs. S8 and S9), but it is unclear if the gene is expressed as trypsin. Using southern blots, Gawlicka and Horn (2006) showed the same *prss* copy number differences between *C. violaceus* and *A. purpurescens*. Ruan et al. (2010) illustrated that vertebrates, fishes in particular, can have all three known *prss* genes (*prss 1*, *prss 2*, and *prss 3*), and that some species express more of one than another. The gene for *prss 3* does not appear to be as strongly expressed in the intestine as *prss 1* and *prss 2* (Castro-Ruiz et al. 2021; Ruan et al. 2010). We found *prss 1* and *prss 2* in the pricklebacks, and both show gut expression (Heras et al. 2020; Herrera et al. 2022). Similar to aminopeptidase activity, *A. purpurescens* has shown roughly doubled the trypsin activity in comparison to *C. violaceus*, on a per-gram tissue basis (German et al. 2004), but not on the whole gut level (German et al. 2015). Perhaps the extra copies of *prss 1* are responsible for the increased trypsin activity in *A. purpurescens*. Interestingly, it was a trypsinogen gene that led to antifreeze glycoproteins in notothenioid icefishes, showing how neo-functionality can arise when there are multiple copies of a gene in a genome, and the dosage effect of the protein isn’t necessary (Chen et al. 1997).

The final protease that showed gene copy number differences between *A. purpurescens* and *C. violaceus* is chymotrypsinogen (*ctr*) and chymotrypsinogen-like (*ctrl*) (Fig. 7, Supplemental Fig. S10). Chymotrypsin is a pancreatic serine protease, like trypsin, but there is some debate as to whether chymotrypsin matters more for fishes consuming more plant material (Gioda et al. 2017; Heras et al. 2020; Ruan et al. 2010; Rungruangsak-Torrissen et al. 2006). Chymotrypsin cleaves different peptide bonds than trypsin does (phenylalanine, tyrosine, and tryptophan as opposed to lysine and arginine for trypsin; Ma et al. 2005), and has shown different temperature optima than trypsin, perhaps providing different activity under different circumstances for an animal (Navarro-Guillén et al. 2022). What is intriguing here is that *C. violaceus* has an extra copy at the *ctrb1* locus, whereas *A. purpurescens* has an extra copy at the *ctrl* locus (Fig. 7, Supplemental Fig. S10). We have never measured the activity of chymotrypsin in the guts of pricklebacks. Chymotrypsin requires more attention to discern its role in the digestive process in fishes consuming different diets (Castro-Ruiz et al. 2019, 2021; Heras et al. 2020; Navarro-Guillén et al. 2022).

In conclusion, we produced a high-quality fish genome, and analyzed it in the context of a growing literature on the nutritional physiology of *A. purpurescens* and other prickleback fishes. Interestingly, the vast majority of sequenced fish genomes are for carnivorous species, since these are largely the ones we culture for human consumption or ornamental use (Heras et al. 2020). In this case, because of the recent publication of the *C. violaceus* genome (Heras et al. 2020), we were able to compare the carnivorous *A. purpurescens* genome to that of a closely related, sympatric, herbivorous species. Based on years of ecological (Horn et al. 1986), physiological (Fris and Horn 1993), biochemical (Chan et al. 2004; German et al. 2016, 2004, 2015), and molecular data (Gawlicka and Horn 2006; Heras et al. 2020; Herrera et al. 2022; Kim et al. 2014), we made a priori predictions about gene copy number for specific digestive enzymes among *A. purpurescens* and *C. violaceus*, and our results largely support the AMH (Karasov 1992; Karasov and Martinez del Rio 2007) from the genomic to the phenotypic level. This powerful physiological genomics approach provides new ways forward in nutritional physiological research, generating new hypotheses on how animals specialize to use different resources (Brun et al. 2020). Indeed, comparative genomics is becoming more common and can lead to more informed understanding of the biology of various taxa, particularly if other data are known about the studied species (Axelsson et al. 2013; Heras et al. 2020; Lamichhaney et al. 2015; Protas et al. 2005; Taylor et al. 2021). Our focus on digestive enzymes is because enzymes are the agents of chemical digestion (Vonk and Western 1984; Karasov and Douglas 2013), and diversity at this key step in nutrient acquisition is likely to inform how animals use various resources (Brun et al. 2020; Heras et al. 2020) although focusing on the liver can also inform about how fishes metabolize various nutrients once they are absorbed (e.g., Heras et al. 2020; Herrera et al. 2022). Finally, given that *C. violaceus* and *A. purpurescens* are commonly found in Marine Protected Areas on the west coast of the United States, our data will also have application for conservation of these species.

### Supplementary Information

Below is the link to the electronic supplementary material.

Supplementary file1 (PDF 1688 KB)

## Data Availability

The final genome annotation was uploaded as project PRJNA950117 at NCBI (https://www.ncbi.nlm.nih.gov/bioproject/).
