# Supplementary material for: The genome of Anoplarchus purpurescens (Stichaeidae) reflects its carnivorous diet
Source: Mol Genet Genomics. 2023 Sep 10;298(6):1419–34. doi: 10.1007/s00438-023-02067-5 (PMC10657299; doi:10.1007/s00438-023-02067-5)
Supplement: Supplementary file 1 — Supplementary file1 (PDF 1688 KB) [file 438_2023_2067_MOESM1_ESM.pdf]

Supplemental Materials for Le et al. (2023) Molecular Genetics and Genomics

Supplemental Table S1. Genome assemblies used for comparisons with *A. purpurescens* and *C. violaceus*.

**Species**	**Genome Assembly**
Danio rerio	GRCz11 (GCA_000002035.4)
Oryzias latipes	ASM223467v1 (GCA_002234675.1)
Gasterosteus aculeatus	BROAD S1
Oreochromis niloticus	O_niloticus_UMD_NMBU (GCA_001858045.3)

Supplemental Table S2. Digestive enzyme genes accessed through Ensembl and or NCBI used for comparison with *A. purpurscens* and *C. violaceus* in this study.

**Species Name**	**Gene Name**	**Ensemble/Gene ID (Gene)**	**Ensemble/NCBI Reference Sequence (Transcript)**
D. rerio	*cel.2*	ENSDARG00000029822	ENSDART00000040598.6
D. rerio	*cel.1*	ENSDARG00000017490	ENSDART00000143952.2
O. latipes	*cel-1a*	ENSORLG00000014439	ENSORLT00000018090.2
O. latipes	*cel-1b*	ENSORLG00000014464	ENSORLT00000018129.2
G. aculeatus	*cel-1*	ENSGACG00000018130	ENSGACT00000024004.1
G. aculeatus	*cel-2*	ENSGACG00000018127	ENSGACT00000024002.1
E. burgeri	*cel*	ENSEBUG00000006718	ENSEBUT00000010991.1
C. idella	*cel-like*	127503333	XM_051876987.1
O. latipes	*cel-like*	ENSORLG00000016428	ENSORLT00000020574.2
H. sapiens	*cel*	ENSG00000170835	ENST00000372080.8
M. salmoides	*cel-1a_1*	LOC119914915	XM_038734457.1
M. salmoides	*cel-1a_2*	LOC119914736	XM_038734219.1
M. salmoides	*cel-like*	LOC119891556	XM_038703291.1
P. olivaceaus	*cel-1a*	LOC109640303	XM_020104213.1
P. olivaceaus	*cel-1b*	LOC109640309	XM_020104221.1
P. olivaceaus	*cel.2*	LOC109640304	XM_020104214.1
T. rubripes	*cel-like*	LOC101064502	XM_003974614.3
T. rubripes	*cel-1a*	LOC115250139	XM_029837300.1
T. rubripes	*cel-1b*	LOC101070169	XM_003978375.3
T. rubripes	*cel.2*	LOC101069939	XM_003978374.3
D. rerio	*ctrb1*	ENSDARG00000090428	ENSDART00000037346.8
D. rerio	*ctrb1-like*	ENSDARG00000093844	ENSDART00000022139.9
D. rerio	*ctrb2*	ENSDARG00000039730	ENSDART00000058067.5

O. latipes	*ctrb1*	ENSORLG00000000391	ENSORLT00000000481.2
O. latipes	*ctrb2*	ENSORLG00000025188	ENSORLT00000035252.1
G. aculeatus	*ctrb1*	ENSGACG00000017663	ENSGACT00000023401.1
G. aculeatus	*ctrb2*	ENSGACG00000017678	ENSGACT00000023415.1
D. rerio	*ctrl-a*	ENSDARG00000088893	ENSDART00000128922.3
D. rerio	*ctrl-b*	ENSDARG00000068680	ENSDART00000099425.4
O. latipes	*ctrl*	ENSORLG00000006426	ENSORLT00000008071.2
G. aculeatus	*ctrl*	ENSGACG00000007594	ENSGACT00000010092.1
P. marinus	*ctrb2*	ENSPMAG00000002029	ENSPMAT00000002258.1
D. rerio	*prss1*	ENSDARG00000042993	ENSDART00000077661.7
O. latipes	*prss1a*	ENSORLG00020002522	ENSORLT00020012963.1
O. latipes	*prss1b*	ENSORLG00020002626	ENSORLT00020012237.1
O. latipes	*prss1c*	ENSORLG00000013582	ENSORLT00000025703.2
G. aculeatus	*prss1a*	ENSGACG00000007461	ENSGACT00000009932.1
G. aculeatus	*prss1b*	ENSGACG00000007422	ENSGACT00000009880.1
G. aculeatus	*prss2*	ENSGACG00000004236	ENSGACT00000005599.1
X. maculatus	*prss1*	ENSXMAG00000010612	ENSXMAT00000010652.2
H. sapiens	*prss1*	ENSG00000204983	ENST00000311737.12
H. sapiens	*prss2*	ENSG00000275896	ENST00000539842.6
D. rerio	*anpepa*	ENSDARG00000041083	ENSDART00000060196.8
O. latipes	*anpepa*	ENSORLG00000019272	ENSORLT00000046140.1
G. aculeatus	*anpepa*	ENSGACG00000002363	ENSGACT00000003088.1
D. rerio	*anpepb*	ENSDARG00000103878	ENSDART00000167730.2
O. latipes	*anpepb*	ENSORLG00020014580	ENSORLT00020021137.1
G. aculeatus	*anpepb*	ENSGACG00000014140	ENSGACT00000018710.1
D. rerio	*anpep Ey*	ENSDARG00000097285	ENSDART00000175736.2

O. latipes	*anpep Ey*	ENSORLG00020014549	ENSORLT00020032697.1
D. rerio	*anpep N a*	ENSDARG00000036809	ENSDART00000172147.3
D. rerio	*anpep N b*	ENSDARG00000089706	ENSDART00000128183.4
O. latipes	*anpep N*	ENSORLG00000014691	ENSORLT00000018415.2
O. latipes	*anpep Ey-like*	ENSORLG00000029229	ENSORLT00000041639.1
G. aculeatus	*anpep Ey-like*	ENSGACG00000014748	ENSGACT00000019527.1
P. marinus	*anpep*	ENSPMAG00000003227	ENSPMAT00000003584.1
P. marinus	*anpep*	ENSPMAG00000009142	ENSPMAT00000010131.1
P. marinus	*anpep*	ENSPMAG00000009172	ENSPMAT00000010133.1
H. sapiens	*anpep*	ENSG00000166825	ENST00000300060.7
R. norvegicus	*anpep*	ENSRNOG00000014610	ENSRNOT00000020002.6
D. rerio	*opn1sw2*	ENSDARG00000017274	ENSDART00000011178.9
G. aculeatus	*opn1sw2*	ENSGACG00000010229	ENSGACT00000013609.1
O. niloticus	*opn1sw2A*	ENSONIG00000020294	ENSONIT00000025592.2
O. niloticus	*opn1sw2B*	ENSONIG00000020293	ENSONIT00000025591.2

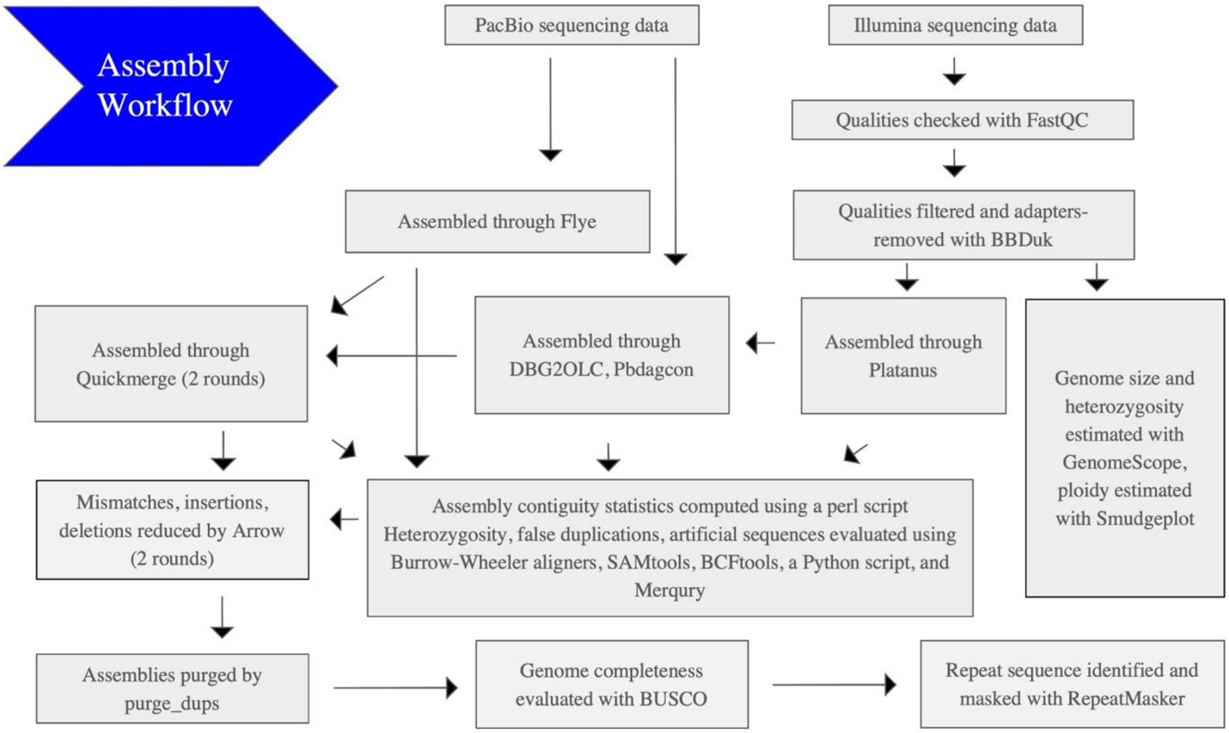

Supplemental Figure S1. Genome assembly pipeline showing the work flow used in this study.

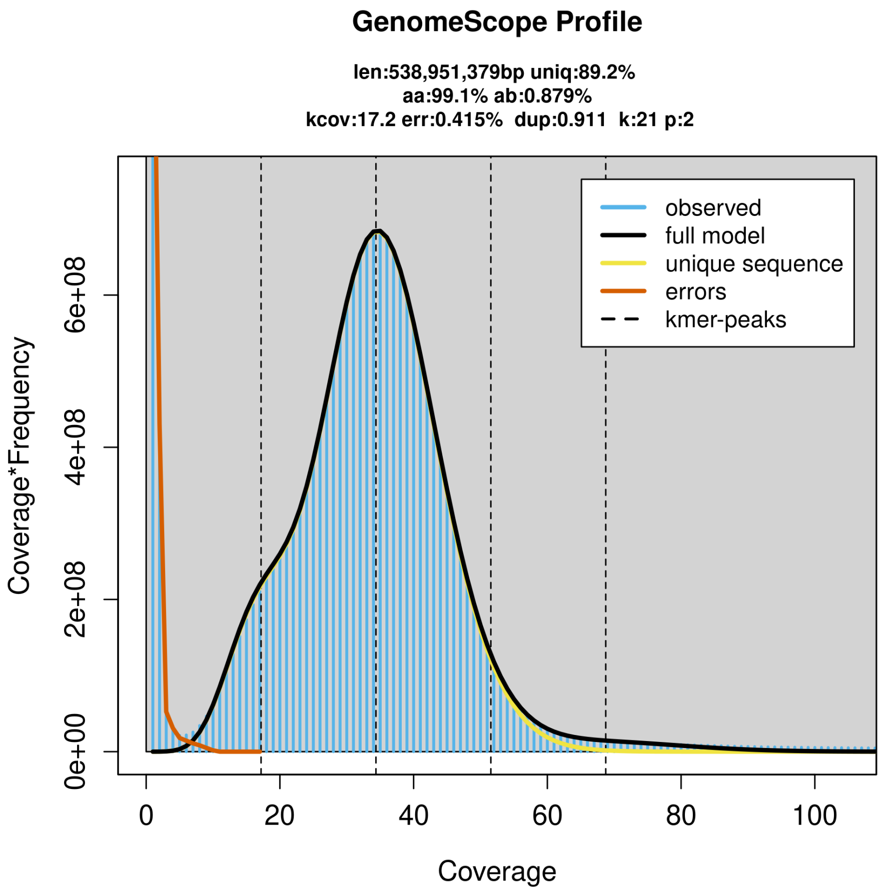

Supplemental Figure S2. K-mer profile plot of the *A. purpurescens* Illumina paired-end sequencing dataset generated by GenomeScope with k-mer size set at 21. Haploid genome length: 538,951,739 bp; Unique k-mers: 89.2%; Heterozygous k-mer coverage: 17.2; Erroneous k-mers: 0.415%; Duplications: 0.911; K-mer size: 21.

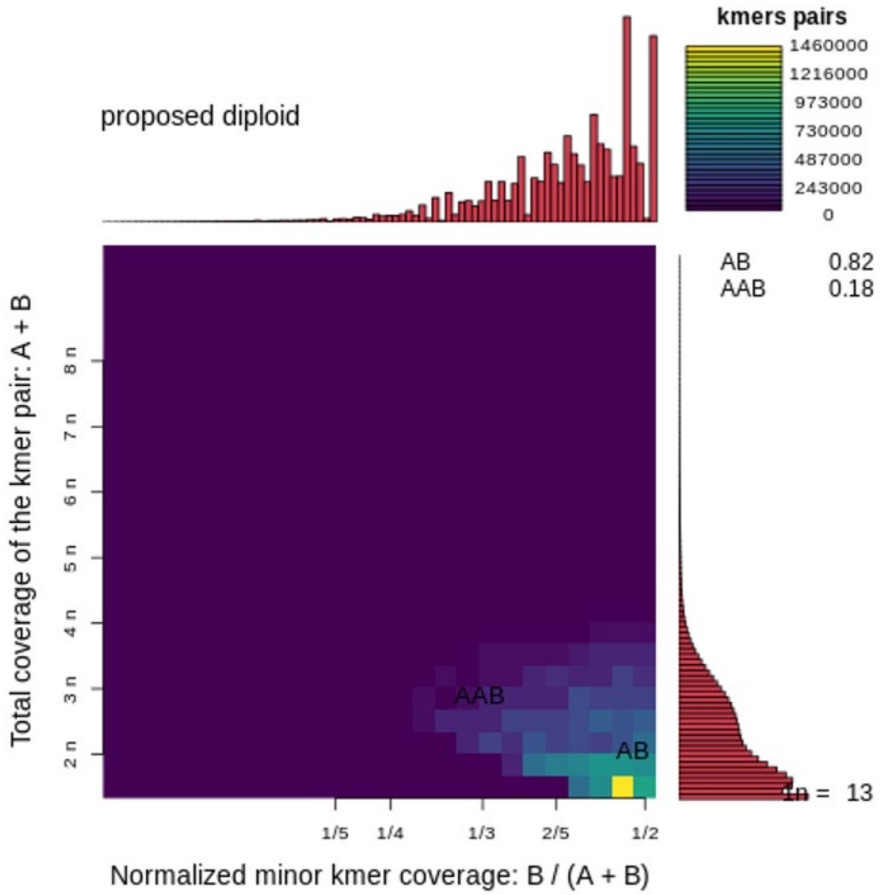

Supplemental Figure S3. Smudgeplot for *A. purpurescens* confirming it is diploid. The plot was generated from *A.purpurescens* Illumina paired-end sequencing data by Smudgeplot and KMC, with k-mer size set at 21.

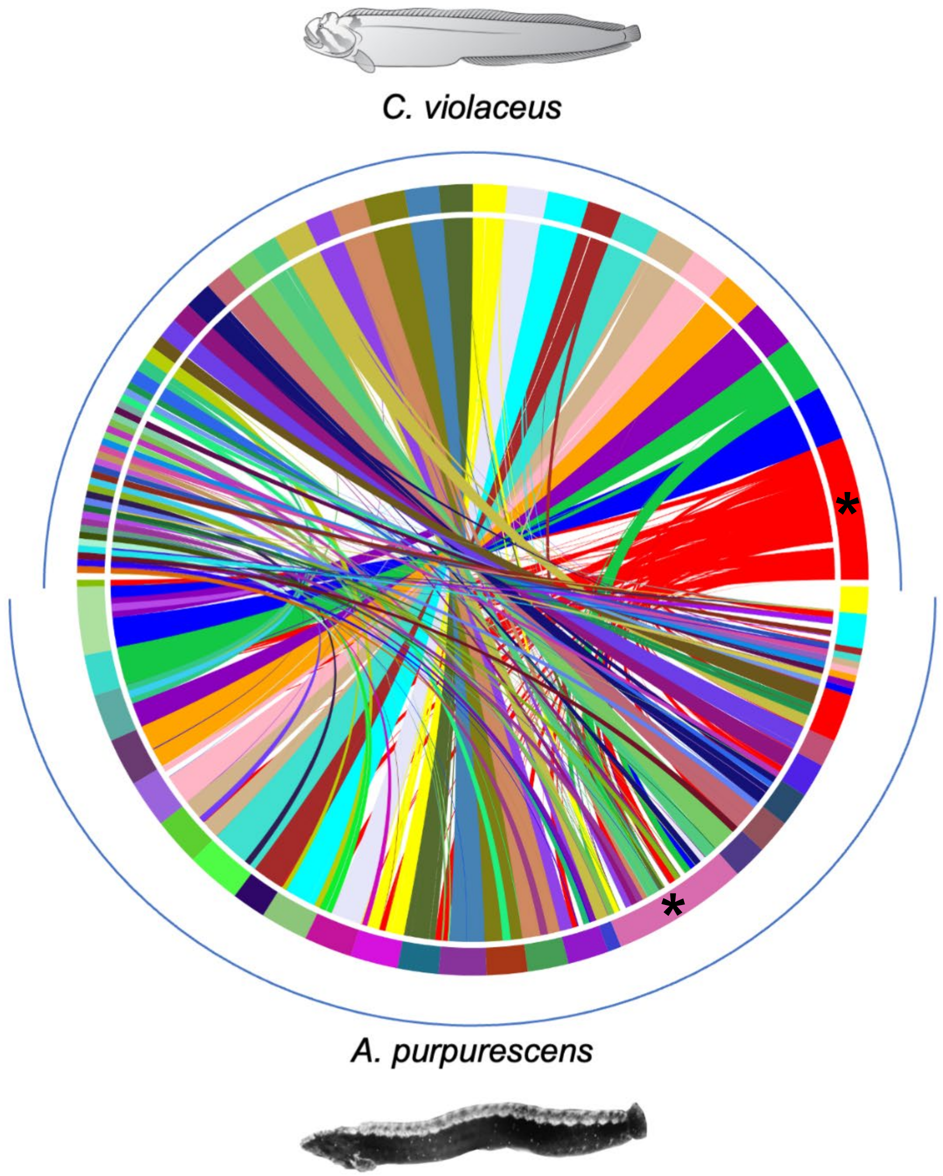

Supplemental Figure S4. Circos plot showing synteny between the assembled genome of *Cebidichthys violaceus* and *Anoplarchus purpurscens*. Each larger block is at least 10Mb in size, whereas contigs smaller than 1Mb were combined into one large contig in each species indicated by an asterick * (red in *C. violaceus*, lavender in *A. purpurscens*). *C. violaceus* illustration was drawn by Andrea Dingeldein, *A. purpurescens* photo by M.H. Horn.

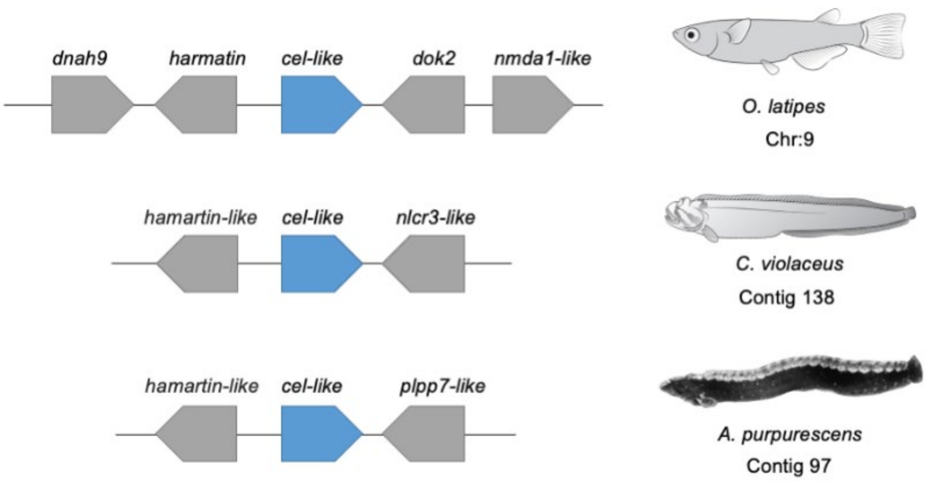

Supplemental Figure S5. Synteny map for carboxyl ester lipase-like genes from *Oryzias latipes*, *Cebidichthys violaceus*, and *Anoplarchus purpurescens*. *O. latipes* illustration was drawn by Andrea Dingeldein.

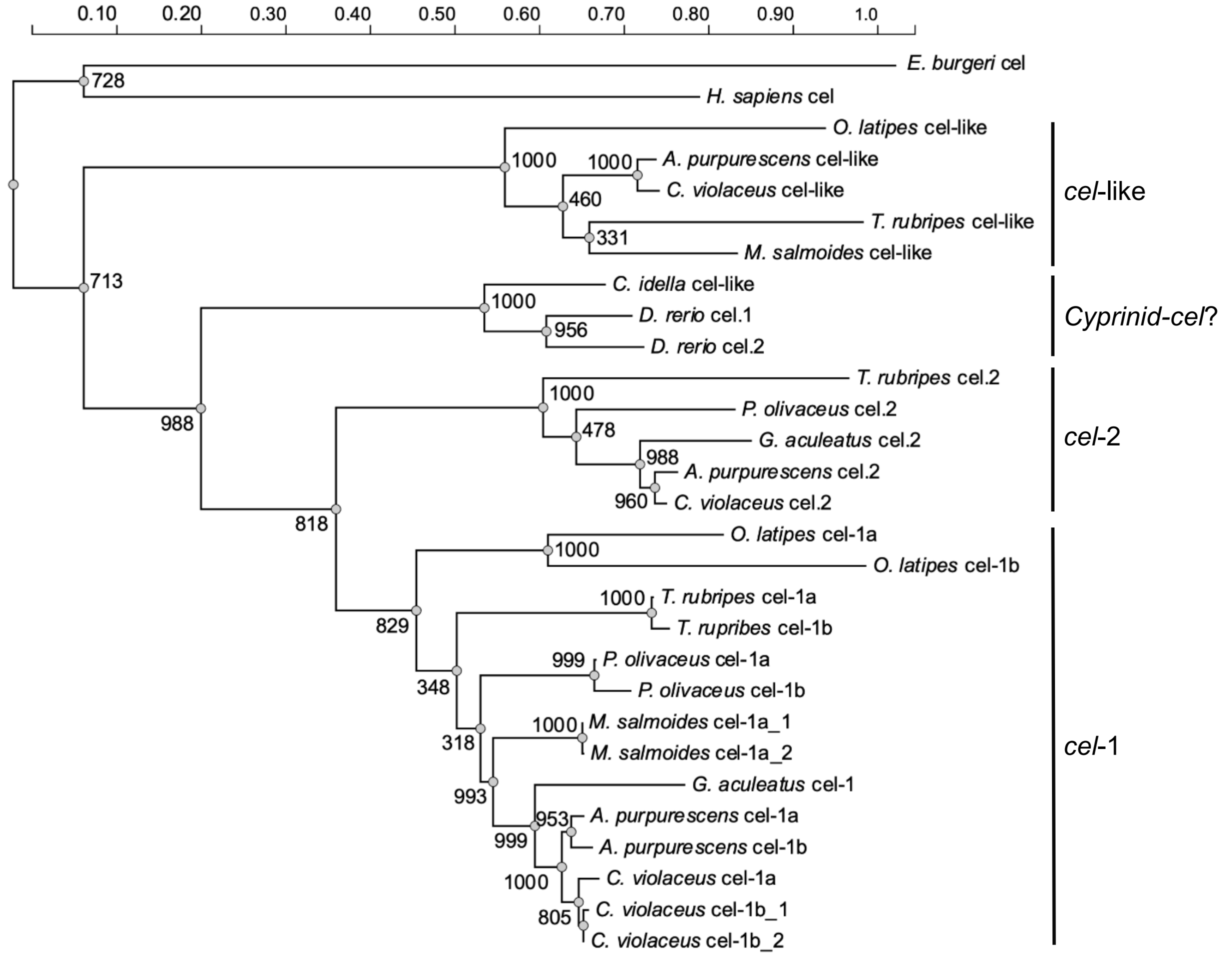

Supplemental Figure S6. Phylogenetic relationship of carboxyl ester lipase (*cel*) genes in fishes (including *Anoplarchus purpurescens*). A maximum likelihood (ML) tree was constructed with 1,000 bootstrap replicates in PhyML v3.0 based on *cel* sequences from *A. purpurescens*, *Cebidichthys violaceus*, *Gasterosteus aculeatus*, *Takifugu rubripes*, *Paralichthys olivaceus*, *Micropterus salmoides*, *Oryzias latipes*, *Danio rerio*, *Eptatretus burgeri*, and *Ctenopharyngodon idella*. *Homo sapiens* and *E. burgeri* formed the outgroups. Note that two cyprinids (*D. rerio* and *C. idella*) have *cel* genes that are sister to all fish *cel*-2 and *cel*-1 genes. A similar gene is also seen in *Cyprinus carpio*, a cyprinid (Tang et al. 2022). Moreover, Tang et al. (2022) showed a similar relationship of these cyprinid *cel* genes not forming a clade with *cel*-like genes, yet being sister to all other fish *cel* genes. Thus, the *cel* genes in cyprinid fishes require more attention since what are currently called *cel*.1 and *cel*.2 in *D. rerio* are not orthologous to the *cel*-1 or *cel*-2 genes of other fishes, at least based on sequence alignment. The *D. rerio cel* genes are syntenic with other fish *cel* genes, however (see Figure 3 in the article).

Tang, S.-L., Liang, X.-F., He, S., Li, L., Alam, M.S., Wu, J., 2022. Comparative Study of the Molecular Characterization, Evolution, and Structure Modeling of Digestive Lipase Genes Reveals the Different Evolutionary Selection Between Mammals and Fishes. Frontiers in Genetics 13.

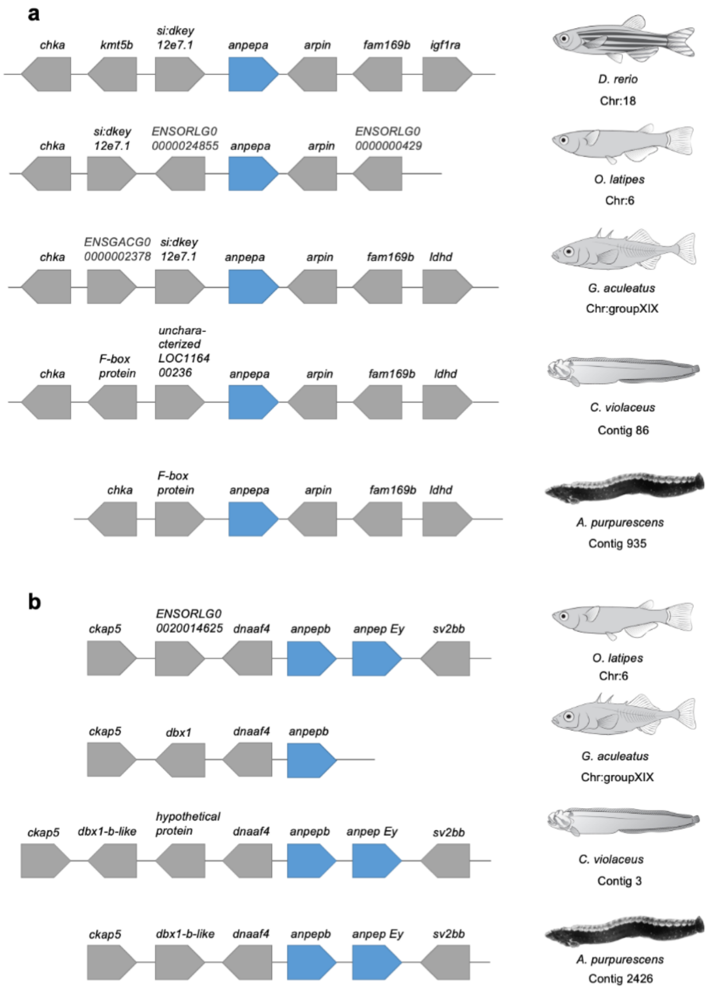

Supplemental Figure S7. a. Synteny map for aminopeptidase a (*anpep a*) genes from *D. rerio, O. latipes*, *G. aculeatus*, *C. violaceus*, and *A. purpurescens*. **b.** Synteny map for aminopeptidase b and Ey genes (*anpep b* and *anpep EY*) from *O. latipes, G. aculeatus*, *C. violaceus*, and *A. purpurescens. D. rerio* illustration was drawn by Andrea Dingeldein.

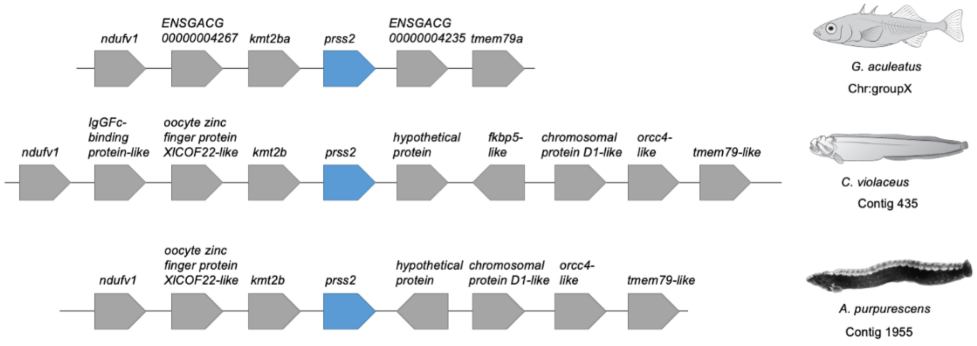

Supplemental Figure S8. Synteny map for trypsin genes (*prss 2*) from *G. aculeatus*, *C. violaceus*, and *A. purpurescens*. *G. aculeatus* illustration was drawn by Andrea Dingeldein.

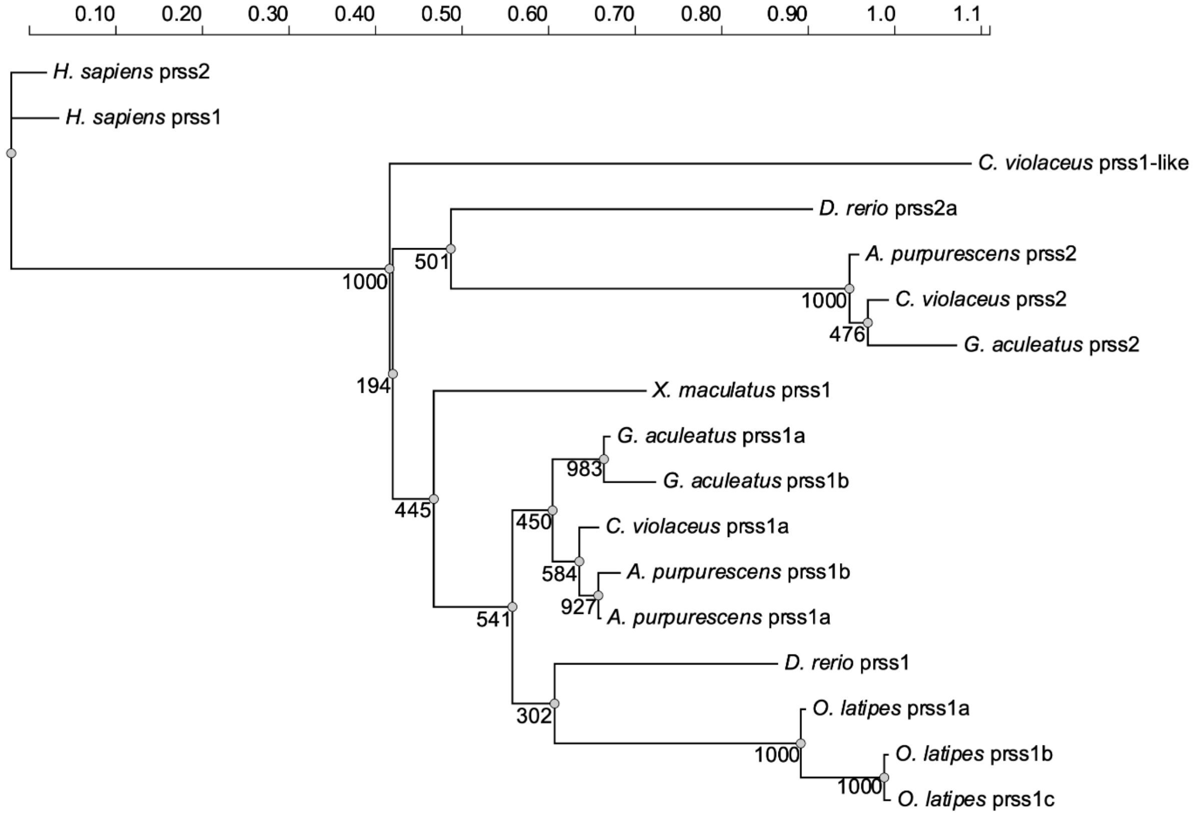

Supplemental Figure S9. Phylogenetic relationship of trypsin genes (*prss*) in fishes (including *A. purpurescens*). A maximum likelihood (ML) tree was constructed with 1,000 bootstrap replicates in PhyML v3.0 based on trypsin sequences from *A. purpurescens*, *C. violaceus*, *G. aculeatus*, *O. latipes*, and *D. rerio*. Trypsin sequences from *H. sapiens* were used as an outgroup.

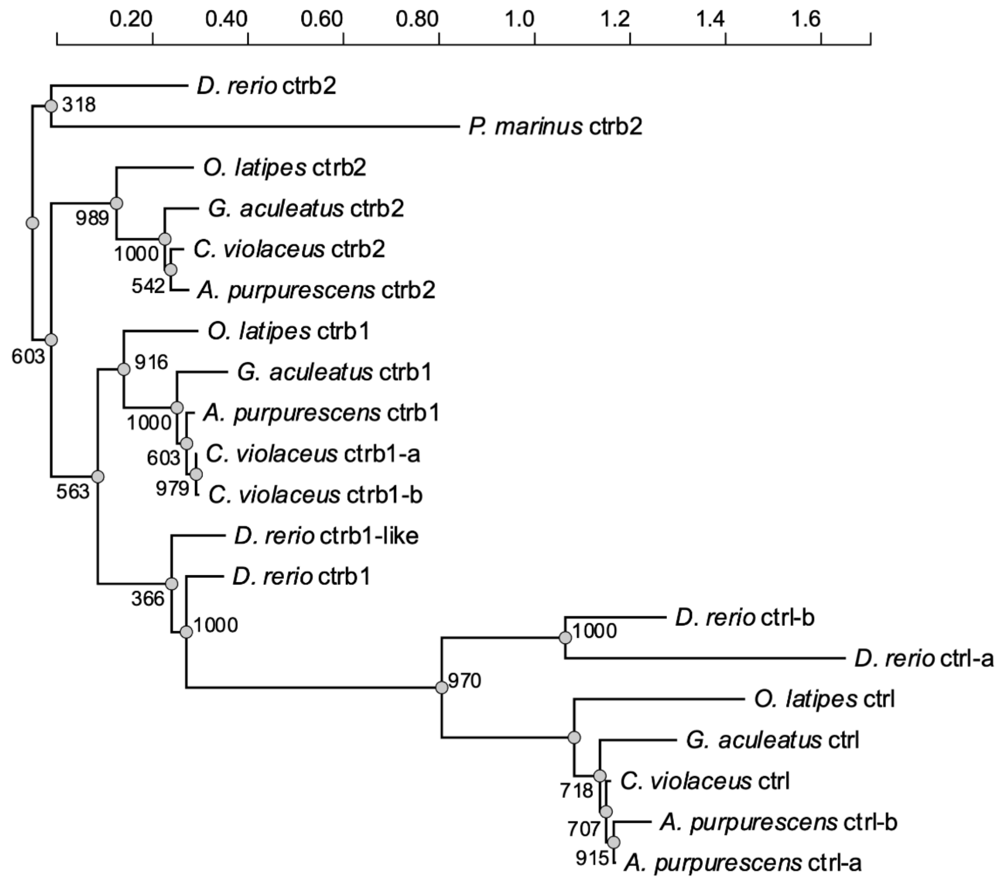

Supplemental Figure S10. Phylogenetic relationship of chymotrypsin (*ctrb*) and chymotrypsin-like (*ctrl*) genes in fishes (including *A. purpurescens*). A maximum likelihood (ML) tree was constructed with 1,000 bootstrap replicates in PhyML v3.0 based on chymotrypsin and chymotrypsin-like sequences from *A. purpurescens*, *C. violaceus*, *G. aculeatus*, *O. latipes*, and *D. rerio*.
